# The College Students’ Oral English Education Strategy Using Human-Computer Interaction Simulation System From the Perspective of Educational Psychology

**DOI:** 10.3389/fpsyg.2021.723981

**Published:** 2021-10-11

**Authors:** Ping Zhou, Xiaoliang Wu, Hui Xu, Guan Wang

**Affiliations:** ^1^School of Foreign Studies, Hunan University of Humanities, Science and Technology, Loudi, China; ^2^School of Foreign Languages, Civil Aviation Flight University of China, Deyang, China; ^3^College of Education, University of Perpetual Help System DALTA, Manila, Philippines; ^4^School of Liberal Arts, Macau University of Science and Technology, Macau, China

**Keywords:** educational psychology, human–computer interaction, simulation, oral English education, educational strategy

## Abstract

The role of the human–computer interaction (HCI) system in college students’ oral English learning is discussed to analyze the current situation of college students’ oral English based on the HCI simulation system. The purpose is to study the oral education of college students. First, the theories of educational psychology, the HCI system, and the current situation of college students’ oral English learning are elaborated. Meanwhile, in oral English teaching, teachers use support vector machines and multimodal fusion intention perception methods in set theory to realize the interactive teaching between students and machines; then, the HCI simulation of oral English is explained. The current situation of college students’ oral English learning is analyzed by a questionnaire from the perspective of educational psychology. Finally, the HCI system in college students’ oral English learning is explored based on the learning level detection. The results show that 12% of college students are unqualified in oral English; 25% of them think their oral English level is medium; most of college students’ English learning anxiety is related to English progress anxiety; 18% of the students believe that they will study oral English for life; 32% of the students think that they have more opportunities to learn English at ordinary times; and most of the students learn English through English movies and songs outside of class. What attracts college students to learn oral English through the HCI system is that learning is not limited by time and space. Most students believe that their English level is good and hope that learning anxiety can be reduced through HCI systems. The strategies of college students’ oral English education with an HCI simulation system are evaluated based on the perspective of educational psychology, providing a research basis for oral English education in other regions and even the whole country to facilitate the better development of oral English education.

## Introduction

The education of college students is closely related to the development of China, and educational psychology is inseparable from education. The development of today’s education industry is no longer limited to the single form of face-to-face classroom teaching. In the field of online education, Massive Open Online Courses (MOOC), interactive classes, live classes, and other forms have come into the public view. For the huge user base in China, the proportion of users covered by excellent teachers and private teachers is very small. At present, in addition to public institutions, China’s training market has fewer than 200,000 teachers, but it has to meet the needs of 230 million users. Among the 200,000 teachers, less than 20% are really recognized and considered “effective” by users. The high cost and long cycle of training excellent teachers make the number of teachers the biggest obstacle to the development of the whole education and training industry (Mohapatra, 2020). In order to solve this problem, technical means and machines need to be used to replace teachers to complete repeated work and standardize the learning process. Copying excellent teachers can break the bottleneck of the education industry, break regional restrictions, and reduce the popularization cost of high-quality teaching resources. The significance of education lies in guiding students to establish correct life values. Teachers inspire and educate students according to the knowledge of educational psychology, improve students’ learning effect, promote students to form positive values, and contribute to the development of education (Wu et al., 2019a).

The research purpose is to integrate educational psychology into a human–computer interaction (HCI) simulation system to help college students’ oral English. The theoretical basis of this exploration is established through the analysis of educational psychology theory, HCI theory, and the current situation of college students’ oral English learning. Then, the students participating in this oral English teaching are investigated and studied through the establishment of a system and the design of a questionnaire. The research innovation aims to realize the education of college students’ oral English through HCI technology, which is a new reform of the traditional education model. It will promote the connection between subject education and computer to a certain extent and improve the quality of teaching (Chen, 2021a).

The above research shows that at present, most college students’ oral English education has changed from the early research on the characteristics of language itself to the current research on artificial intelligence, oral learning, and education. With the continuous development and renewal of HCI and college students’ oral English education, intelligent language learning products under the application of new technologies such as artificial intelligence continue to promote the development of oral English education. In this context, from the perspective of educational psychology, the HCI simulation system strategy is innovatively applied to college students’ oral English education. The impact of an HCI simulation system on college students’ oral English education strategy is studied from the perspective of educational psychology through the analysis of the application of interactive simulation system, the model mechanism of the interactive system, and the questionnaire on the educational practice of oral applied learning so that a better oral education goal is achieved. This exploration is aimed at college students’ oral education, but it can also be applied to other disciplines to lay the foundation for the development of intelligent education.

## Literature Reviews

From the perspective of educational psychology, the method of applying an HCI simulation system to college students’ oral English teaching has become a research topic of many scholars.

Souza et al. (2020) proposed an HCI teaching process, which combines traditional lecture-based classrooms (TLBC), active learning, and project-based learning to improve students’ understanding of HCI. The background of this is academic courses in undergraduate scientific computing include theoretical courses and practical courses. Theory courses involve more abstract content. This process aims to increase students’ participation in HCI theory. Different from Souza, Chen (2018) analyzed the advantages of constructing a simulation teaching system based on HCI virtual reality technology, summarized it from the functional level, and reflected on the traditional classroom. In addition, the application of virtual reality technology in HCI in the development of simulation teaching system platform and the functional aspects that need to be improved in design research were mainly analyzed to help comprehensively improve the operation effect of the system and promote the better application of virtual reality technology in the design of teaching system. However, the research on HCI in the fields related to English teaching was not specifically discussed. Wang (2019) found that the seewo all-in-one machine can complete real-time writing and real-time erasure, and multiple people can write at the same time. A relaxed HCI situation is an effective means to promote the human–computer interactive teaching of English in senior high school. Therefore, the theme of “the application of HCI (teachers and students, network, seewo all-in-one machine, etc.) teaching in senior high school English teaching” was explored. Hu et al. (2020) conducted further research on this, and they found that there are many factors affecting English classroom teaching, and teachers cannot effectively grasp students’ learning. In particular, classroom management in network teaching is more difficult. In order to improve the effectiveness of English classroom teaching, the HCI process and classroom learning of students in network teaching are effectively recognized based on the HCI algorithm and face recognition algorithm, and the image background is removed according to the actual teaching needs. Moreover, the spatio-temporal feature image that can express dynamic typical features is obtained by extracting and fusing the spatio-temporal information of motion in the video. Perera and Liyanage (2021) found that brain–computer interface technology has been widely used in education, autonomy, development, marketing, safety, sports and entertainment, heart reading, telecommunications, and other fields, and it has been used in treatment and rehabilitation. Among the existing brain–computer interface technologies, the brain–computer interface technology based on EEG is still the most popular, surpassing some invasive and non-invasive technologies. The research of Xu et al. (2020) is more detailed. They believed that an electrooculogram (EOG) is a bioelectrical signal recorded by an electrode and formed by the potential difference among eyeball, retina, and cornea; the barrier-free HCI system controlled by EOG plays an increasingly important role in serving patients with limb movement control disorders. They summarized the establishment and development of EOG controlled HCI system; introduced the common methods of eye movement detection: search coil method, infrared eye movement diagram method, Purkinje image tracking method, and the method based on computer graphics and EOG; and summarized the application of EOG controlled HCI system in medical assistance for the disabled and operation in a dangerous environment. Finally, the limitations of the HCI system controlled by EOG and its future development trend are analyzed.

To sum up, the development of the HCI simulation system has been relatively mature. However, the application of the HCl simulation system in college students’ oral English education is still in the early stage of development. The research advantage is to study the application of the HCI simulation system in college students’ oral English education from the perspective of educational psychology, which is a novel perspective. This exploration is related to promoting the quality of oral English education, and the related research literature is relatively lacking. This exploration plays an expanding and complementary role in the research of related fields. Moreover, the research is of great practical value, which can help students understand oral English and provide students with a good language simulation environment.

## Experimental Design and Research Process

### Educational Philosophy From the Perspective of Educational Psychology

The teaching method is relatively fixed in the traditional teaching mode, which basically includes classroom teaching and homework. It cannot play the role of educational psychology and impart the knowledge of educational psychology thoroughly. Educational psychology and education are inseparable. The significance of education lies in guiding students to establish correct life values. Teachers enlighten and educate students according to the knowledge of educational psychology to improve students’ learning effect, promote students to form positive values, and boost the development of education. In recent years, educational psychology has been introduced into network education, and the electronic platform has been applied to teaching to make students learning through various online learning. From the perspective of educational psychology in the new era, strengthening the shaping of school physical education personality is a crucial platform and channel for students to build self-expression. Moreover, students need to correctly understand and treat themselves in a certain way in the process of daily learning and social life. Meanwhile, this understanding will be adjusted with the development of the situation. This phenomenon is called “self-expression” from the perspective of educational psychology. Meanwhile, students often encounter the following situations in their daily life. That is, some students prefer to express themselves, while some students do not (i.e., whether individuals are willing to show their advantages in front of students). Some students are willing to attract the attention of other students in any case and especially hope to leave a good impression on the students. The research results of domestic theoretical circles show that some scholars and researchers believe that this kind of self-expression can be called “self-presentation” or “self-display.” This statement is similar to “self-expression” or “impression management” in English. Some western scholars regard self-expression as a behavior to give full play to and expand the image on the stage of self-life. The people who are willing to perform self-expression will take various reasons to promote self-expression and further convey the connotation closely related to their personal image. It belongs to the category of individual internal psychology and the research focus of social educational psychology. It reveals that both western self-expression and eastern self-expression can effectively use the shaping function of physical education personality from the perspective of educational psychology. It is embodied in fully perceiving the impression of others at the same time and hoping to form a good impression of others as a social individual. Thereby, school physical education based on the perspective of educational psychology has a far-reaching impact on students’ physical and mental health development and education, which cannot be replaced by other similar courses and disciplines.

Wu et al. (2019b) evaluated the application of social media in online course learning, believing that it not only enriches teaching methods but also helps to broaden students’ horizons. The combination of educational psychology and network under the background of network education can increase multiple learning methods that are not available in traditional teaching mode, shorten the distance between students and educational psychology, and promote the development of China’s education.

### Theory of HCI Simulation System

HCI is the process by which people get information through the output device of a computer and control the computer through the input device. Generalized HCI covers hundreds of interaction modes (Gurcan et al., 2021). Although people expected HCI to develop in the direction of virtual reality technology and so on as early as the end of the 20th century, the most popular and most available way today is still the graphical user interface (GUI) and mouse keyboard input (Wu et al., 2020), and the GUI will not be replaced. It has been proposed that we examine the emotional labor of team members and investigate the mechanisms adopted by these teams to improve their members’ emotional contagion levels. Hence, the HCI behavior considered here mainly refers to the method based on the WIMP (window, icon, mouse, and pointer) interface in a narrow sense, but the correlation analysis and modeling method should be able to provide a reference for the implementation of touch screen interaction, voice interaction, and other similar ways (Yy et al., 2021). The HCI simulation system studies the coordination relationship between humans, computers, and the environment. According to different conditions and characteristics, it uses physiology, psychology, and other related knowledge (Jung et al., 2020) to reasonably allocate the operation functions undertaken by humans and computers and makes them adapt to each other. It can create a safe, convenient, and comfortable working environment for people and make the work efficiency reach the best.

#### Speech Recognition Module

In speech recognition technology, the client software uses two kinds of technology: one calls the Baidu voice (API) online, and the other is there as backup support. A Microsoft voice API on a local machine will be called only when network partition occurs. The Baidu voice API is powerful, accurate, and easy to call. It does not need to be installed locally by users. A general Microsoft voice API is native to win7 and above operating systems, but it may not have its own voice library in some compact operating systems. Users need to install the appropriate version by themselves, which is cumbersome to use. Hence, it is only used as backup support.

Baidu voice API is free and open to developers. To use the functions of Baidu voice, first, users need to apply for a key in the open platform of Baidu voice, download the Software Development Kit (SDK) related to Baidu voice, and complete the necessary configuration. Then, the dynamic link library needs to be imported into the client software.

#### Action Recognition Module

**Figure 1:**
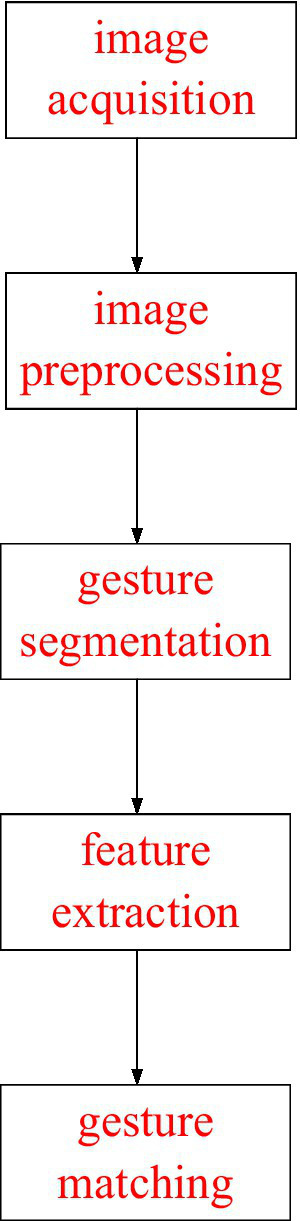
Action recognition process.

At present, there are many ways to realize gesture recognition. The specific implementation of the gesture recognition system proposed mainly consists of five parts: image acquisition, image preprocessing, gesture segmentation, feature extraction, and gesture matching, as shown in Figure 1.

First, a camera is employed to capture the video data stream. In essence, the video data stream includes a frame-by-frame image sequence. Then, the image is preprocessed to eliminate the noise and enhance the quality of the image; next, the color space of the image is transformed, and an appropriate skin color model is established to judge whether there are gestures. If a gesture is detected, gesture segmentation is carried out. The geometric feature parameters of the current gesture are extracted based on the analysis of the separated gesture, and then gesture recognition is carried out by template matching.

### Design of Multimodal Fusion Intention Perception Method System Based on SVM and Set Theory

#### Hardware Design

A simulation experiment system of multimodal fusion intention perception method based on support vector machine (SVM) and set theory (MSSN system) is adopted. First, the system allows users to use VR (virtual reality) equipment for the experiment to cultivate teachers’ application ability in the actual teaching process. Meanwhile, the immersion of the oral English virtual teaching environment is improved by using simulation equipment and natural interaction methods such as oral English and gestures. Then, the user’s interaction intention is perceived through the multimodal information sent by the user in the process of the experiment to make up for the lack of intelligence of the virtual experiment and bring about a better interactive experience for the user. Finally, navigational interaction is adopted to navigate for users to reduce the load of users and improve the participation of teachers and students in the teaching process (Hu et al., 2020).

1. Therefore, regarding the hardware, VR glasses, Bluetooth scanning handle, smartphone hardware equipment, high-performance server, and high-capacity storage equipment are selected. The selection follows the principles of advanced technology, reasonable economy, applicable in the course, as well as the requirements of feasibility, maintainability, operability, and energy supply to determine the optimal scheme of the equipment
2. Selection of VR glasses: after wearing the VR head display, users put a mobile phone into the box as a display and computing device and can see the images of the left and right parts with the help of the left and right eyes, respectively. The brain can process two images and produce stereo vision. With the help of a mobile phone accelerator and gyroscope, the head posture of the human body is determined. At this time, users can transfer their perspective by moving their head so they can initially experience the charm of virtual reality technology and bring students an immersive feeling
3. Selection of Bluetooth scanning handle: the Bluetooth scanning handle can be an ordinary handle
4. Smartphone selection: there are no specific requirements for the selection of mobile phones, just ordinary smartphones. Any version of Android and IOS can meet the operation of the platform
5. Hardware environment; the system is required to report the operation environment, so an ordinary PC can meet the operation requirements.

#### System Structure

**Figure 2:**
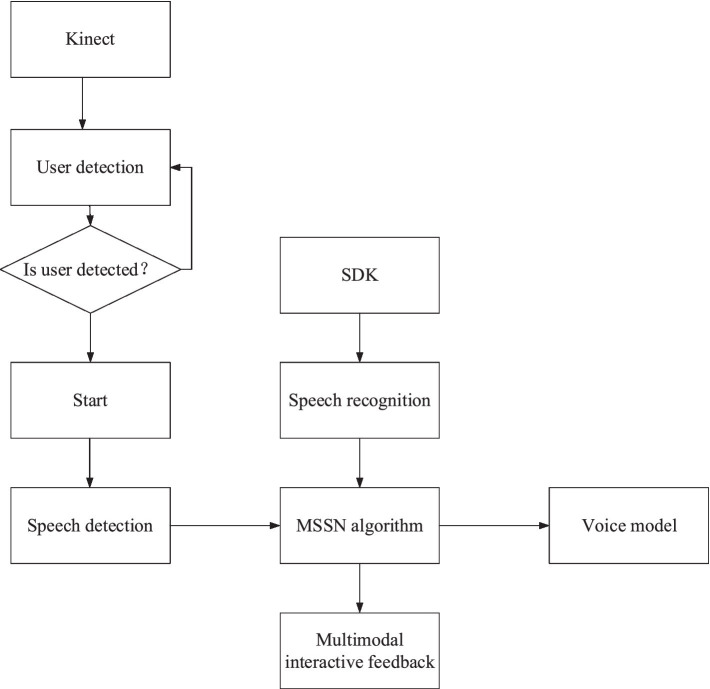
System architecture.

**Figure 3:**
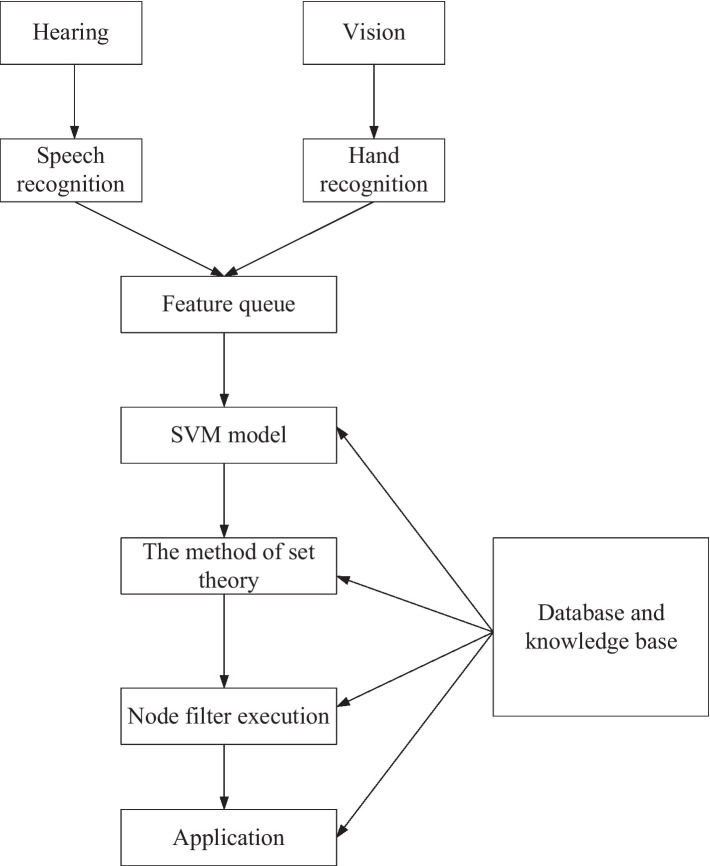
MSSN algorithm.

Figure 2 shows the structure of the SVM and MSSN simulation experiment system. First, the system detects whether there are users according to the user information obtained by the kinect device. Then, the system uses a kinect device to recognize the position of human hands and uses Baidu Speech SDK for speech recognition and sentence segmentation to get speech recognition results. The voice information of touch and vision can be obtained through the VR device. The recognized visual, auditory, and tactile information is communicated *via* the MSSN method. Users’ complex operation intention can be perceived according to the multimodal fusion algorithm. Finally, according to the perceived user intention, the system carries out multimodal interaction with users, including visual feedback, experimental equipment feedback, and voice navigation, which greatly improves the intelligence of the system. The student voice recognized by Kinect is applied to the virtual reality model in the virtual scene. MSSN algorithm and LBP algorithm are employed to recognize the students’ voices and emotions, realize the interaction with the machine, and sense the students’ emotions in real time. Figure 3 presents the structure of the MSSN algorithm.

The MSSN algorithm is divided into four layers: multimodal input layer, recognition layer, multimodal fusion intention perception layer, and application layer. The core is the multimodal fusion intention perception layer. In this layer, multimodal data are organically combined to form the form of intention behavior nodes, which is convenient for the computer to understand students’ more complex intentions.

#### System Environment

Software environment: Windows10, Unity 2018, Visual Studio 2015, Baidu voice;

Programming language: C#.

#### System Test Scheme

In total, 41 sophomores and 12 English teachers from a college in Xi’an were invited to experience the experimental system to evaluate the MSSN system proposed. For the same experiment, the experimental results are compared and analyzed by using the smart oak intelligent English system and the MSSN system. The full score in the experiment is 5. The questionnaire includes the following aspects (Zlatkovic et al., 2020). The student part contains interesting, exploratory, and learning mastery; and the teacher part contains students’ interest in learning, an explanation of key contents, and students’ participation.

### Research on the Current Situation of College Students’ Oral Education

There is a growing number of studies on oral English anxiety in recent years. Most students’ oral anxiety level is higher than that of listening, writing, reading, and other skills, and oral anxiety is relatively difficult to overcome (Chen et al., 2021). It is urgent to study how to reduce oral anxiety to improve students’ comprehensive foreign language level and promote effective communication (Kirova, 2020; Tsai et al., 2020).

In oral English learning, it is difficult for college students to have the opportunity to use English in addition to communicating in English class, leading to students’ lack of enthusiasm, self-confidence, and a poorer language environment for learning English. The college students in China are deeply influenced by Chinese and word-to-word translation thinking, which leads to poor oral English expressions. There is a big gap in western English expression, but the gap is also narrowing. There are differences in the way of oral expression, sentence structure, and the meaning of words. Chinese students often blame their poor oral English on their insufficient vocabulary and then recite dictionaries aimlessly. In fact, this is not an effective way to learn oral English. The oral polysemy needs to be found and mastered in language use. Most of them come from life, not from high-level words.

### Research Route of Oral English Education for College Students

**Figure 4:**
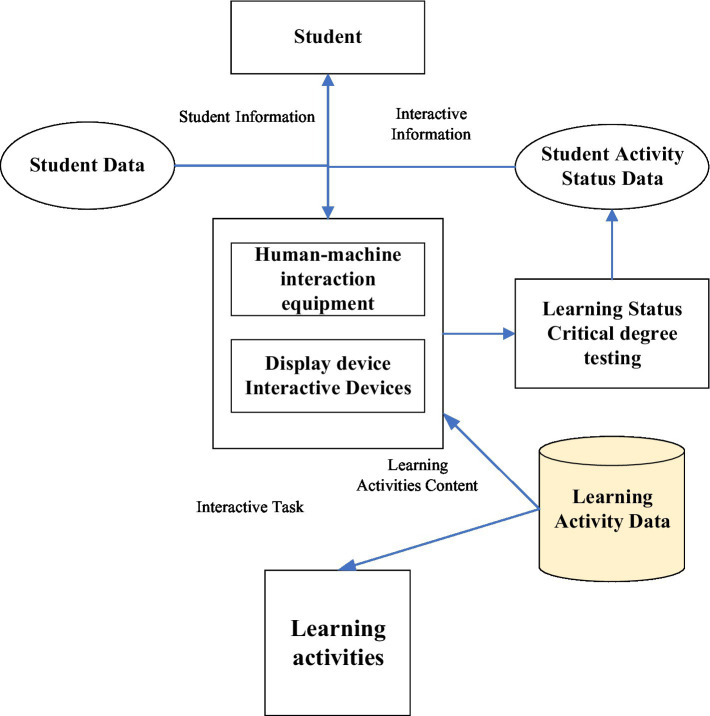
Structure of HCI learning system.

**Figure 5:**
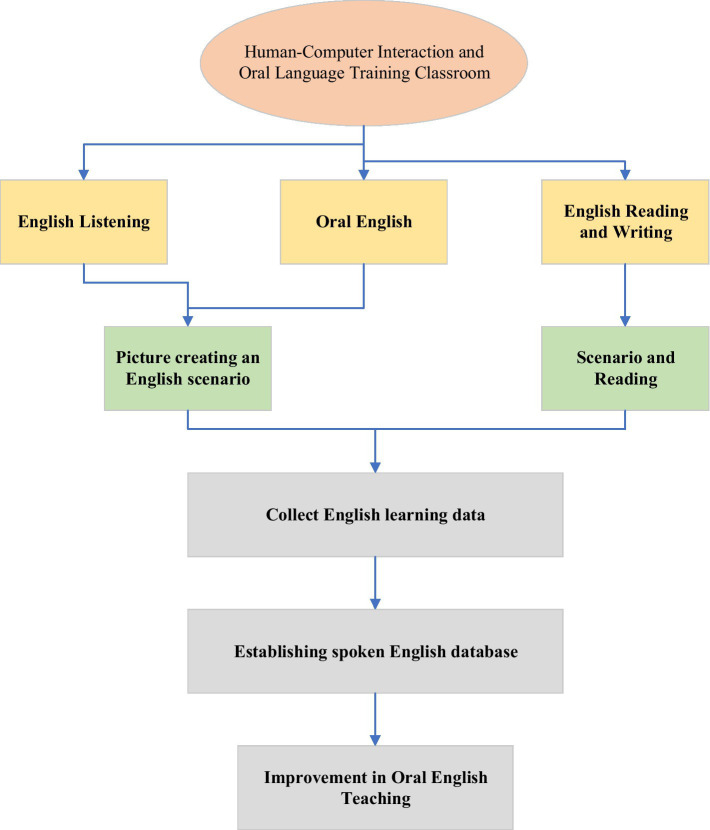
Flow chart of HCI oral training course.

College students’ English education has always been a troubling issue, and the state attaches great importance to it. The structure diagram of the human–computer system shows the relationship among each module in the HCI learning system intuitively (Jian et al., 2020). Teachers can query students’ information and learning status information through HCI equipment. Student information comes from the student database, and learning status information comes from the learning status database (Choi and Park, 2020). Figure 4 displays the structure of the HCI system. Figure 5 is the flow chart of the HCI oral training course (Wu et al., 2020). Students can also obtain learning activities through HCI equipment, and the information of learning activities comes from the learning activity database (Chen, 2021b). Learning status data are generated when students conduct HCI learning, including interest level test results, students’ learning activities, learning time, and achievements, which are stored in the learning status database (Qian et al., 2018).

## Questionnaire Design and Model Selection

### Questionnaire Design

The questionnaire was chosen to study the management of college students’ oral English teaching under the HCI simulation system from the perspective of educational psychology. The questionnaire is primarily distributed through the “XX” platform, mainly for sophomores and juniors. Teachers of the college students’ entrepreneurship center are contacted in advance before the questionnaire is issued to ensure the effective recovery of the questionnaire (Wu and Song, 2019).

Overall, 500 questionnaires are distributed, and 450 questionnaires are obtained by statistics, with an effective rate of recovery of 90%. SPSS is adopted to analyze the reliability and validity of the questionnaire. It is found that the questionnaire has good reliability and validity. The questionnaire aims to analyze the situation of college students’ oral English. Table 1 is a few key issues involved in the questionnaire design.

**Table 1:** Key question design of the questionnaire.

The questions are as follows:	Content and specific options	Score
Q1: What is your self-evaluation of oral English?	M11: excellent	10
	M12: good	8
	M13: medium	6
	M14: pass	5
	M15: fail	3
Q2: What is the motivation for learning oral English?	M21: learning interest	10
	M22: under the pressure of study	3
Q3: What kind of oral English teaching do you like?	M31: Traditional teaching	3
	M32: Interactive teaching	6
Q4: Do you have multiple opportunities to speak English?	M41: There are many opportunities to learn oral English	10
	M42: There are few opportunities to learn oral English.	5
Q5: Do you agree with the idea of learning English for life?	M51: agree	8
	M52: disagree	5
	M53: relatively agree	6
Q6: Do you have any other ways of learning oral English besides studying in class?	M61: English movies	6
	M62: English songs	6
	M63: English club	3
	M64: English reading and other ways	4
Q7: Is there anxiety in oral English learning?	M71: Yes	2
	M72: No	8
Q8: What is the current anxiety level of students?	M81: serious	5
	M82: slight	10
	M83: no	15
Q9: What do you think is the source of oral English anxiety?	M91: progress anxiety in class	20
	M92: classroom speaking anxiety	20
	M93: evaluation anxiety	20
	M94: oral anxiety	20

### An Analysis of Oral Education Strategies

Oral English education for college students is the key direction of national education. The calculation method of Harr characteristics is introduced (Li, 2020) based on the questionnaire to analyze college students’ interest in oral English education and learning and further analyze the current situation and development of college students’ awareness of learning oral English. A machine learning algorithm is adopted to obtain the face data of students’ learning, and face recognition is carried out by the detection machine (Liu et al., 2020). Then, the inner corner of the human face is labeled and taken as the level, and processing is performed at a distance of 50 pixels. The equation is as follows:


(1) $$\left\{\begin{array}{c} N=\frac{50}{x1-x2}\\ R=\arctan(\frac{x1-x2}{y1-y2} \end{array}\right.$$

N is the scale of the image, R is the rotation angle of the image, and (x1, y1) and (x2, y2) are the coordinates of the left and right inner eye corners, respectively.

The basic LBP algorithm of expression recognition is as follows:


(2) $$LBP\left(A1,B1\right)=\sum_{m=0}^{m=1}2mS\left(Lm-Ln\right)$$

(A1, B1) is the center pixel of the selected area, m is the number of other pixels except for the center pixel of the selected area, and the brightness is Ln; Lm is the brightness of adjacent pixels. S is a symbolic function:


(3) $$S\left(x\right)\left\{\begin{array}{c} 1\;if\;x\geq 0\\ 0\;else \end{array}\right.$$

After the calculation of the above equations, the machine can identify the psychological anxiety of students in learning oral English and the psychological changes after learning through the HCI system and collect the information of students learning oral English to assess the situation of students learning English. Chen et al. (2021) proposed that the HCI system can judge other people’s emotional changes according to the results of emotion recognition. The experimental software used is the data analysis software SPSS 25. version. The operating system of the system software running environment is win10 with a 64-bit processor. The CPU model is Intel Core i7 11700K, the solid-state hard disk is 1T, the mobile hard disk is 2T, the GPU model is 8GB LPDDR4, and the system running memory is 8G. The questionnaire is conducted on the “College Students Forum” platform, which is a national online communication platform with college students as the main service object.

## Experimental Results and Performance Evaluation

### Analysis of Simulation Results

**Figure 6:**
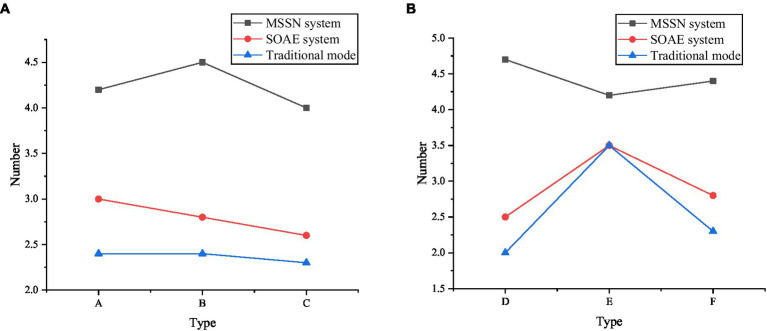
Test results of oral English teaching in knowledge learning [**(A)**: students; **(B)**: teachers; A: Interest; B: Exploratory; C: learning mastery; D: students’ interest in learning; E: key content explanation; F: student participation].

Figure 6 displays the experimental results of oral English teaching in knowledge learning in two systems. The data image above reveals that compared with the traditional teaching methods (video and PPT) and SOAE English intelligent teaching system, the MSSN system shows a good data effect in learning English knowledge. First, for college students who have not learned the intelligent teaching mode, it can stimulate their interest in the HCI mode, make them better understand knowledge and have a deep impression on oral English knowledge, and better cultivate their ability of inquiry learning through intelligent perception. According to the statistics of English teachers’ teaching data: through teachers’ intuitive operation and systematic phenomenon feedback, it is more conducive to the teaching of key English contents, stimulates students’ interest in learning, and cultivates students’ participation to enhance students’ understanding of oral English.

### Reliability and Validity Analysis of Questionnaire

**Figure 7:**
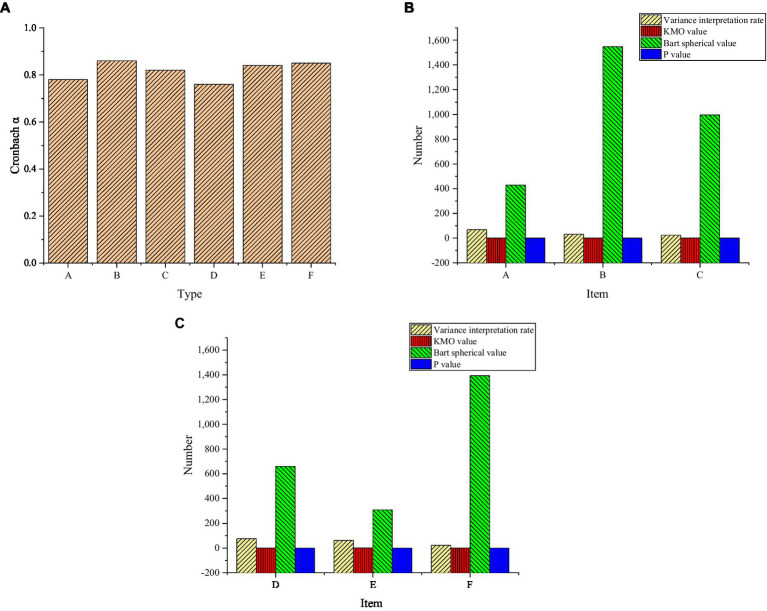
Reliability and validity analysis of questionnaire content—**(A)** reliability analysis results of the questionnaire; **(B)**: validity analysis of content variables in the questionnaire; **(C)**: validity analysis of teacher content variables in the questionnaire; A: Interest; B: Exploratory; C: Learning mastery; D: Students’ interest in learning; E: Key content explanation; F: Student participation.

Figure 7 below presents the reliability and validity analysis of the questionnaire. The reliability coefficient of each factor in figure (A) shows that Cronbach’s α coefficient statistics of interest, learning mastery, key content explanation, and student participation are higher than 0.8 and have good reliability. The Cronbach’s α coefficient of exploration and students’ interest in learning is in the range of 0.7–0.8, which also meets the reliability requirements. The KMO values of interest, exploration, learning mastery, students’ learning interest, key content explanation, and students’ participation are greater than 0 and close to 1, and there is thus a good correlation among the factors. Moreover, in the chi-square value of Bartlett’s test, it is found that the *p* values are less than 0.05, and they have thus reached the significant level. Therefore, the above factors can be used for exploratory factor analysis.

### Analysis of Questionnaire Survey Results

**Figure 8:**
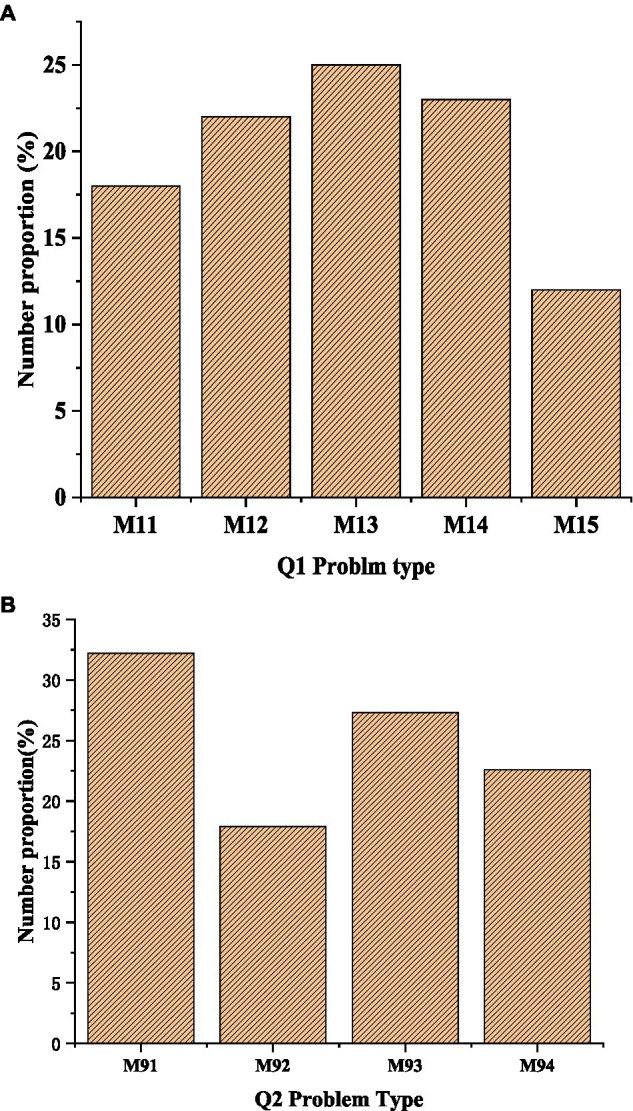
Survey results based on Q1 and Q9: **(A)** the survey results based on Q1; **(B)** the survey results based on Q2.

The survey results of several key items in the questionnaire are analyzed. Figure 8 below shows the survey results based on Q1 and Q9. Figure 8A shows that in Q1, 12% of college students are unqualified in oral English; 25% think their oral English level is medium; Figure 8B shows that regarding Q9, English progress anxiety M91 accounts for the largest proportion of 32%, classroom speaking anxiety M2 accounts for the smallest proportion of 16%, and oral self-confidence accounts for a slightly higher proportion than classroom anxiety, indicating that college students hope that a better oral English learning environment can be provided and HCI system classroom can be set up, that is, a classroom interaction model of internet and oral English teaching.

**Figure 9:**
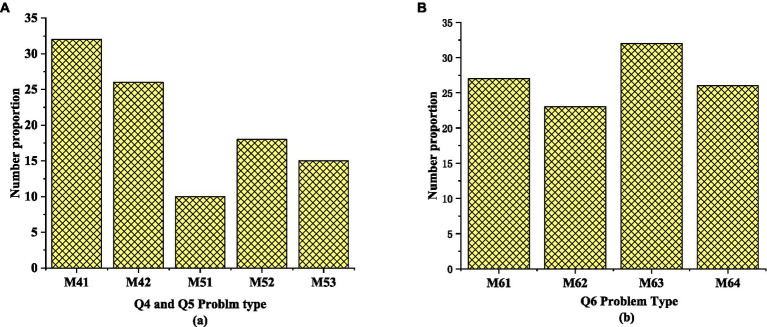
Survey results based on Q4-Q6: **(A)** the survey results of Q4 and Q5; **(B)** the survey results of Q6.

Figure 9 shows the survey results based on Q4-Q6. Figure 9A suggests that M41 accounts for 32% of the students’ oral English learning opportunities, M52 (disagree with the idea of lifelong learning English) accounts for 18% of the students, and 8% of the students agree with lifelong learning oral English, indicating that college students are more willing to accept oral English learning. The number of students who choose English clubs to learn oral English is up to 33%, and the number of students who choose English movies and other ways to learn English is the same, that is, the proportion of M61 and M64 is the same (6%).

The above results demonstrate that college students have a great intention to learn oral English in the HCI system. However, oral English training is limited by time and space. Regarding HCI oral training system, students can download the corresponding app software on their mobile phones. Whether it is in the classroom, playground, or other places, as long as they carry a mobile phone, students can carry out oral training at any time and will not be disturbed by others. Wu et al. (2018) believed that this kind of information and communication technology (ICT) is employed to improve the effectiveness and ability training of traditional teaching methods. This free learning space can effectively alleviate students’ anxiety in learning English in class; besides, the HCI system has the function of privacy protection (Li and Chen, 2021). Every time the study is completed, there are corresponding practice results, and students can see their advantages and disadvantages. With repeated practice and learning, students’ anxiety about oral English learning will gradually reduce. Most students are optimistic about the HCI system of oral English teaching, indicating that the HCI simulation system has a certain role in promoting college students’ oral English teaching.

Table 2 displays the statistical results of the whole scale.

**Table 2:** Statistical results of the overall scale.

Item	Number of students	Average value	Standard deviation	Minimum value	Maximum
The whole scale	500	67.5	12.3	35	86

The researchers analyzed the survey data to study the psychological situation of college students’ oral English before the implementation of oral training. In Table 2, regarding the whole scale, the average score of students’ oral English learning is 67.5, and the standard deviation is 12.3. According to the classification standard of Oxford & Burry-Stock (1995), if the average score of each item is less than 2.4, the level is relatively low; if the average score is much higher than 3.5 (including 3.5), it indicates that the level is relatively high. Besides, if the average value is between 2.5 and 3.4, the descriptive statistical results of the overall scale in Table 2 show that the average value of the overall scale is 67.5, and the standard deviation is 12.3. The average score of each item is 2.93, which is between 2.5 and 3.4, and this can lead to the conclusion of medium level. Before the teaching experiment, the level of college students’ oral English anxiety is above the middle level. Besides, the standard deviation of 12.3 shows that there are great individual differences in college students’ oral English anxiety.

**Figure 10:**
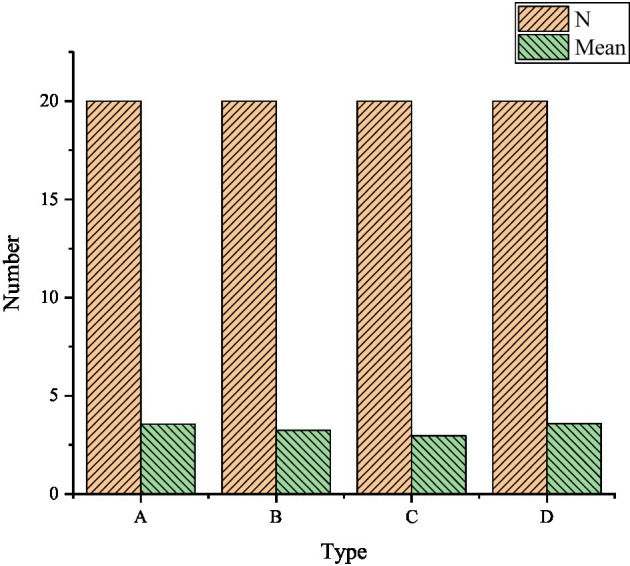
Descriptive statistical results of English anxiety types—A: classroom progress anxiety; B: classroom oral anxiety; C: evaluation anxiety; D: oral anxiety.

Moreover, a separate statistical analysis on the types of English anxiety is made, and each dimension of the scale is analyzed. Figure 10 presents the descriptive statistical results of English anxiety types.

Descriptive statistical results of English anxiety types in Figure 10 show that oral anxiety of college students has the highest average score (*M*=3.58) and evaluation anxiety has the lowest score (*M*=2.96), indicating that oral communication anxiety is the main source of oral anxiety (Solomon, 2021). The most important function of language is that it is a tool for human communication (Rl et al., 2020). In the current education and examination environment, oral English education has been greatly affected.

### Comparison of the Effect of Oral English Training Before and After the Use of the HCI System

**Figure 11:**
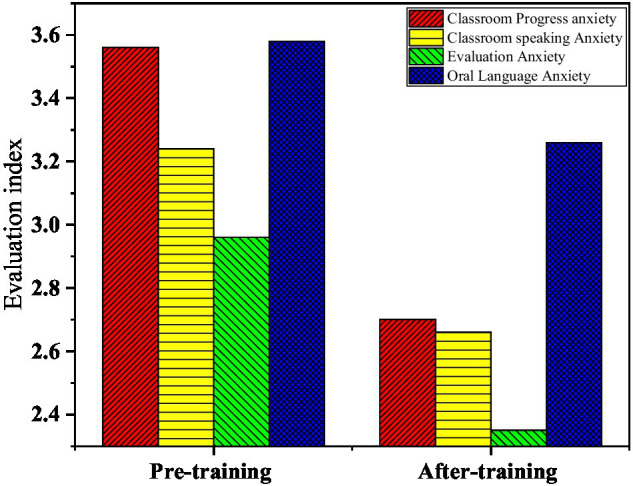
Comparison before and after training.

**Figure 12:**
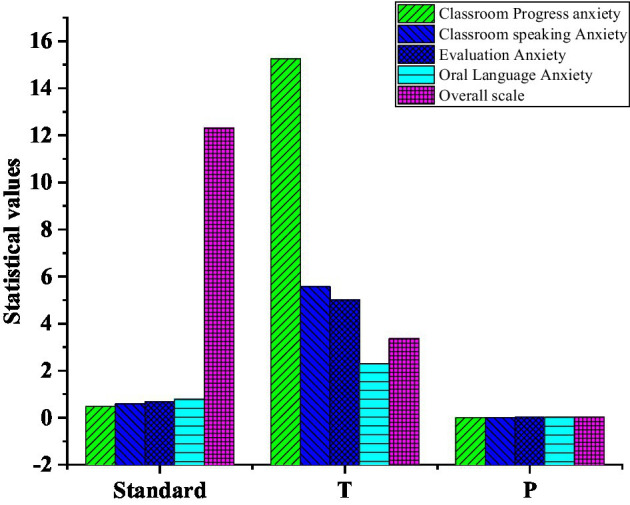
Result chart of correlation analysis: standard stands for standard deviation; T is the T value of correlation analysis; value of *p* indicates the degree of correlation.

In Figure 11, the comparison results before and after the training show that the four kinds of anxiety after the training are reduced to varying degrees, indicating that the use of the HCI system in oral English learning is helpful and reduces the students’ psychological pressure and anxiety in learning oral English. Figure 12 suggests that there are significant differences in four dimensions in the paired sample *t*-test, including classroom progress anxiety (*p*=0.000<0.05), classroom speaking anxiety (*p*=0.000<0.05), evaluation anxiety (*p*=0.017<0.05), and oral anxiety (*p*=0.025>0.05). The above data once again proves that the HCI training system is an effective way to improve learners’ learning motivation (Fang et al., 2021) and self-confidence. Researchers conduct paired sample t-test on the data (Matheson and Petersen, 2020) to explore the specific role and influence of the HCI system on college students’ oral English learning psychological state. In the overall scale, there is a significant difference (*p*=0.000<0.01) in the psychological level of oral English before and after the implementation of the HCI system (Bergstrm and Hornbk, 2019), and the average score of the sample decreases from 67.5 to 57.8. The HCI oral training system effectively intervenes in the college students’ oral English state (Farinde and Omolaiye, 2021).

## Discussion

The above results show that the MSSN system shows a good data effect in learning English knowledge compared with traditional teaching methods (video and PPT) and SOAE English intelligent teaching system. According to the statistics of English teachers’ teaching data, teachers’ intuitive operation and systematic phenomenon feedback are more conducive to the teaching of key English contents, which can stimulate students’ interest in learning and cultivate students’ participation to enhance the students’ understanding of oral English. College students have a great willingness to learn oral English in the HCI system because this learning method has no time and space constraints. Better privacy protection function reduces students’ psychological anxiety in oral learning and plays a positive role. The research data and analysis results support the research purpose and design idea of this exploration. Unlike many studies in the previous literature review, the application of the HCI simulation system in college students’ oral English education is studied from the perspective of educational psychology. The questionnaire data analysis is fully demonstrated, and the research results meet the expected objectives.

## Conclusion

The strategy research of college students’ oral education based on the HCI simulation system from the perspective of educational psychology shows that college students should also focus on their psychological state when taking oral education. Teachers should make the English classroom lively and interesting, and integrate the HCI system into oral English education. Then, students should avoid outdated English learning habits to adapt to the new mode of oral English education.

The psychological factors and current situation of college students’ oral English learning are evaluated *via* a questionnaire and the method of simulating the test before and after the training of oral English. The results reveal that the average score of the subjects to learn oral English is 67.5 and the standard deviation is 12.3. The HCI training system is an effective way to improve the self-confidence and learning motivation of learners. The calculation method of Harr characteristics is introduced. The students’ anxiety in learning oral English is evaluated by a machine learning algorithm along with face data and oral English training data. HCI oral simulation learning can effectively intervene in the psychological state of college students based on the perspective of educational psychology. The sample selection is limited because of the influence of the actual research conditions, which cannot cover all aspects. The improvement of oral English ability is a gradual process. Hence, according to the results, it is suggested that college students should not be in a hurry during oral English education. The cultivation of oral English ability is a long process and does not happen overnight. The limited time in class can only meet the needs of the students’ basic knowledge, and the cultivation of practical oral communication ability still needs to be carried out in daily life. Therefore, it is very crucial to guide students to cultivate their oral English ability independently in their daily life. Teachers should consciously establish this awareness in the teaching process and guide students from the following two aspects. On the one hand, in daily life, teachers should take the initiative to improve their oral English communication ability, influence students’ learning concepts with practical actions, and imperceptibly implant the consciousness of self-training oral communication ability into students’ consciousness; on the other hand, teachers should focus on students’ psychological state in daily life, affirm and encourage students who try to communicate in oral English, eliminate students’ fear of English communication, and cultivate students’ awareness of independent training.

However, there are still some limitations. The sample selection is limited due to the influence of the actual research conditions, which is not enough to cover all aspects. The improvement of oral English ability is a gradual process. College students should have good learning habits and perseverance, and teachers need to practice and explore new and effective teaching methods. In the future study, college students should consciously cultivate their own oral thinking, improve their oral fluency, and actively participate in social practice to improve the effectiveness of oral learning and adapt to the rapid development of English in the future.

## Data Availability Statement

The raw data supporting the conclusions of this article will be made available by the authors, without undue reservation.

## Ethics Statement

The studies involving human participants were reviewed and approved by Hunan University of Humanities, Science and Technology Ethics Committee. The patients/participants provided their written informed consent to participate in this study. Written informed consent was obtained from the individual(s) for the publication of any potentially identifiable images or data included in this article.

## Author Contributions

All authors listed have made a substantial, direct and intellectual contribution to the work, and approved it for publication.

## Funding

This work is supported by Research Project of Teaching Reform in Colleges and Universities of Hunan Province (2021).

## Conflict of Interest

The authors declare that the research was conducted in the absence of any commercial or financial relationships that could be construed as a potential conflict of interest.

## Publisher’s Note

All claims expressed in this article are solely those of the authors and do not necessarily represent those of their affiliated organizations, or those of the publisher, the editors and the reviewers. Any product that may be evaluated in this article, or claim that may be made by its manufacturer, is not guaranteed or endorsed by the publisher.
